# Recent Advances in Gene Therapy for Hemophilia: Projecting the Perspectives

**DOI:** 10.3390/biom14070854

**Published:** 2024-07-15

**Authors:** Nikita Chernyi, Darina Gavrilova, Mane Saruhanyan, Ezekiel S. Oloruntimehin, Alexander Karabelsky, Evgeny Bezsonov, Alexander Malogolovkin

**Affiliations:** 1 Laboratory of Molecular Virology, First Moscow State Medical University (Sechenov University), Moscow 119435, Russia; black.0513@mail.ru (N.C.); manesaruhanan954@gmail.com (M.S.); olorunsolaezek@phystech.edu (E.S.O.); 2 Department of Biology and General Genetics, First Moscow State Medical University (Sechenov University), Moscow 105043, Russia; dzibrova@yandex.ru; 3 Center for Translational Medicine, Sirius University of Science and Technology, Sochi 354530, Russia; karabelskiy.av@talantiuspeh.ru

**Keywords:** hemophilia, gene therapy, immune tolerance induction, viral vectors, CRISPR-Cas-9, bispecific antibodies, AAV

## Abstract

One of the well-known X-linked genetic disorders is hemophilia, which could be hemophilia A as a result of a mutation in the *F8* (factor VIII) gene or hemophilia B as a result of a mutation in the *F9* (factor IX) gene, leading to insufficient levels of the proteins essential for blood coagulation cascade. In patients with severe hemophilia, factor VIII or factor IX activities in the blood plasma are considerably low, estimated to be less than 1%. This is responsible for spontaneous or post-traumatic bleeding episodes, or both, leading to disease complications and death. Current treatment of hemophilia relies on the prevention of bleeding, which consists of expensive lifelong replacement infusion therapy of blood plasma clotting factors, their recombinant versions, or therapy with recombinant monoclonal antibodies. Recently emerged gene therapy approaches may be a potential game changer that could reshape the therapeutic outcomes of hemophilia A or B using a one-off vector in vivo delivery and aim to achieve long-term endogenous expression of factor VIII or IX. This review examines both traditional approaches to the treatment of hemophilia and modern methods, primarily focusing on gene therapy, to update knowledge in this area. Recent technological advances and gene therapeutics in the pipeline are critically reviewed and summarized. We consider gene therapy to be the most promising method as it may overcome the problems associated with more traditional treatments, such as the need for constant and expensive infusions and the presence of an immune response to the antibody drugs used to treat hemophilia.

## 1. Introduction

Hemophilia has been studied since ancient times, from the papyri of ancient Egypt to the second century AD, as discovered in the Talmud’s text [1]. The disease was nicknamed the kings’ disease by nineteenth-century scientists, as this was common in the royal dynasties of Europe [1]. Symptomatically, the disease is often identified by sudden bleeding episodes in the sufferers [2]. In severe cases, FVIII and FIX concentrations in the plasma are extremely low and often associated with disease complications and death [3,4].

The incidence of hemophilia A and B globally is estimated at 1:5000 and 1:30,000 newborns, respectively; the incidence rate is higher among boys than girls [4]. Thus, it can be expected that the number of hemophiliacs globally may be over 1.1 million, among which about 400 thousand are likely to develop serious cases of hemophilia [5].

**Figure 1:**
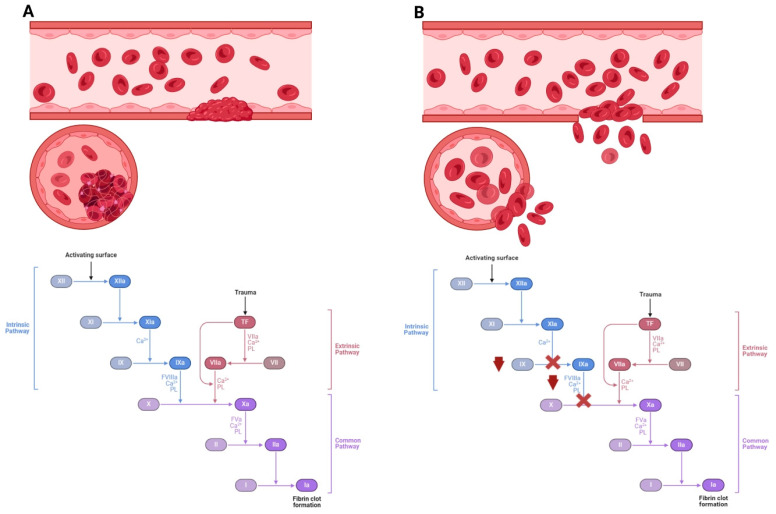
The incidence of hemophilia disease as caused by impaired blood clotting factors. (**A**) Coagulation cascade and blood clot formation with the physiological level of factor VIII and factor IX. The disease is caused by low levels of coagulation factors in the blood. (**B**) With a lack of factor VIII, type A hemophilia develops, while a lack of factor IX leads to type B hemophilia. Activated FVIII and FIX activate factor X, thereby increasing thrombin synthesis from prothrombin. A lack of factor VIII or IX leads to reduced thrombin formation. Hemophilia may cause spontaneous bleeding and death.

Currently, the most common treatment for hemophilia B is bleeding prevention, which consists of expensive lifelong infusion therapy with plasma clotting factors or recombinant factor IX. Even in developed countries such as the USA, Germany, Japan, etc., the cost of blood clotting factors to treat one patient with hemophilia B can reach USD 450,000 per year, and the lifetime treatment costs eventually reach USD 20 million or more [2]. In developing countries, on the contrary, the situation is worse as patients generally lack the opportunity to receive such supportive therapy, at least regularly, and face high chances of exsanguination (fatal and high blood loss) and poor quality of life due to the development of ankylosis and contractures. The Figure 1 below represents factors involved in hemophilia and its development.

Gene therapy can be considered the most promising method for treating hemophilia A and B. According to the European Association for Haemophilia and Allied Disorders (EAHAD), eleven clinical trials for hemophilia A and B are ongoing with more than 300 patients exposed to the gene therapy treatment (202 patients for hemophilia A and 135 for hemophilia B) (https://eahadgtd.mdsas.com/ (accessed on 24 June 2024)). The desired implementation of this treatment approach is a one-time vector injection, which leads to constant endogenous production of factor FVIII or IX. The target therapeutic dose is considered to be an FIX level that is 1% higher than the normal value; in practice, this significantly reduces the likelihood of bleeding.

There are several reasons why gene therapy could be a more desirable option for hemophilia treatment. Even a very small increment in the expression of these clotting factors can have a huge impact on the overall condition of patients. The levels of the plasma factor can be measured to assess gene expression. Early studies using an ex vivo gene therapy approach through gene transfer in hematopoietic stem cells or autologous fibroblasts resulted in transient low expression of FVIII [6]. The approach used in early studies by transferring genes ex vivo on hematopoietic stem cells or autologous fibroblasts resulted in transient expression of FVIII. Over 20 years ago, recombinant adeno-associated virus (AAV)-based therapy was introduced to administer FIX (rAAV-FIX) to people with hemophilia B through intramuscular administration. This method was deemed considerably safe and provided patients with over three years of expression of coagulation factors. Unfortunately, this could not improve the expression of FIX at the required concentrations, as the level of this factor remained below 1% in most patients [7,8,9]. Modern drugs enable the stable expression of coagulation factors for extended periods exceeding eight years [10].

In recent studies, the success rate of therapy for hemophilia based on current immune tolerance induction (ITI) protocols has been reported to be around 60% to 80% depending on several factors such as age at ITI start, race or ethnicity, FVIII genotype, and historical inhibitor peaks influencing the outcomes [11,12]. However, ITI is unsuccessful for 20–40% of patients with severe hemophilia A and a larger percentage of patients with mild hemophilia of both types [13]. Also, unfavorable factors for the onset of ITI are a high peak inhibitor titer in children, older adult patients for whom the formation of a new inhibitor is traced in 20–50 days of ITI, older adult patients with inhibitors present for a long time who did not receive a test ITI and patients with previous unsuccessful ITI [14]. Moreover, the costs of ITI therapy type remain relatively high.

Despite ongoing improvements to various gene therapy approaches, challenges remain with immunogenicity, expression efficiency, vector reliability, genotoxicity, and persistence. Also, there is a problem of ineligibility of children to benefit from gene therapy for hemophilia due to considerations related to liver development and the durability of transgene expression [12]. During product development of gene therapy, it is crucial to consider patient selection criteria, adopt a multidisciplinary approach to follow-up, and develop optimal pricing policies and financial reimbursement models for patients.

This review examines both traditional approaches to the treatment of hemophilia and modern methods, including gene therapy, to update knowledge in this area and track recent progress and challenges. Here, we provide a short overview of available treatments for hemophilia and an outlook on promising next-generation gene therapies. The search of the literature was conducted using the PubMed database and keywords such as “hemophilia” AND “gene therapy”, OR “therapy of hemophilia”. This therapeutic approach remains the top choice due to its ability to overcome the problems associated with traditional treatment methods. These include the need for constant expensive infusion procedures and the presence of an immune response to the antibody drugs used against hemophilia.

## 2. The Treatment of Hemophilia Using Blood-Purified Factors VIII and IX

In the 1950s and 1960s, whole blood plasma transfusions were the main form of treatment for hemophilia. However, the amount of required clotting factors it contained was insufficient, necessitating long-term infusions of large volumes of donor plasma in a hospital setting to achieve positive results from the procedures.

Cryoprecipitation technology emerged in the second half of the 1960s, allowing for the precipitation of necessary blood proteins under a specific thawing regime for fresh frozen plasma. In this manner, it is possible to obtain concentrated clotting factors, significantly reducing infusion volume. The obvious way to treat hemophilia when the molecular actors of this disease were identified was to purify factors VIII and IX from donor blood to administer these factors to patients.

Cryoprecipitation was the first step in obtaining FVIII from plasma, followed by the extraction and aluminum hydroxide precipitation procedure. The last stage of this process involves either immunoaffinity chromatography using monoclonal antibody immobilization or ion exchange. Ion exchange chromatography does not remove von Willebrand factor (vWF), whereas immunoaffinity chromatography results in pure FVIII. However, the resulting product is not stable and requires the addition of serum albumin [15]. Chromatographic purification is complicated by the complex structure and high sensitivity to proteolysis of FVIII [15]. The purification of these coagulants could also be achieved by the use of affinity chromatography [16], but the quality of the resulting products in terms of additional protein contamination significantly limited their effectiveness.

To prevent viral contamination of purified plasma-derived factor concentrates, they undergo pasteurization and detergent treatment. The resulting products are classified based on the ratio of FVIII to other proteins, namely medium purity, highly purified, and ultrapure.

However, the conventional method of treatment can be burdensome for patients, particularly young children, as it necessitates constant intravenous injections and incurs high financial costs. Additionally, inhibitor antibodies that attach to non-functional epitopes of FVIII, rendering the product inactive, can form and pose difficulties. Studies indicate that the risk of developing inhibitory antibodies is influenced by genetic factors such as gene deletion or nonsense mutation on FVIII, as well as ethnicity. To obtain clotting factors, it is necessary to combine a large number of donor plasma samples, which increases the risk of infection transmission. Up to 60–70% of individuals with severe hemophilia were found to have contracted HIV in the 1980s, and nearly all of them contracted hepatitis C [17]. Following the discovery, more rigorous measures were implemented in the decontamination of donor plasma samples. These measures included the use of detergents, pasteurization, and temperature treatment. However, despite these measures, the likelihood of pathogenic agents penetrating the plasma was not reduced to zero. This led to an increased demand for the development of safer methods for obtaining the necessary coagulation factors. The technology of expressing recombinant proteins from mammalian cell cultures was crucial [17]. For instance, emicizumab [18,19], a bispecific antibody mimetic of FVIII and FIX, enhances the action of FIXa and FX, thereby increasing thrombin generation. However, there are contradictions to this issue, as some researchers believe that recombinant factors’ usage increases the chances of inhibitor formation. When deciding between recombinant and plasma-derived treatment options, it is important to consider the immunophenotype of the patient (a group of distinct markers or antigens found on the cell’s surface, nucleus, or cytoplasm), and the tendency to generate inhibitory antibodies must be taken into account. “International recommendations on the diagnosis and treatment of acquired hemophilia A” suggest refraining from the use of recombinant or plasma-derived human FVIII concentrates if bypassing agents or rpFVIII are available or ineffective and the inhibitor titer is low [20].

## 3. Treatment of Hemophilia Using Recombinant Factors VIII and IX

Recombinant FVIII was first to be engineered in 1982, and then in 1984, FIX was successfully cloned. The first recombinant protein-based medications were created in the 1990s. BeneFIX, a licensed recombinant FIX medication, was made accessible in 1997 to treat hemophilia B [17,21].

It is important to note that blood clotting factor preparations are biological drugs, which means that the body may develop immunological reactions to them. In clinical practice, if such cases arise, the drug should be discontinued and decongestants prescribed. Additionally, confirmatory immunological tests for IgE should be considered to determine the appropriate therapy. In case of severe hemophilia in patients with factor IX administration, anaphylactic reactions and inhibitors to this factor may occur. In a study of two patients with severe hemophilia B using a skin test and RAST factor IX reset, as well as subsequent desensitization to factor IX, it was found that IgE causes anaphylactic reactions in response to factor IX administration [22].

As recombinant factor VIII (rFVIII) preparations were developed over the years, they were classified based on the proteins used in their production, either animal or human, to reduce immunogenicity [23].

Recombinant FVIII products can be classified based on their source of production, either from animal or human cell culture. The earliest products used animal proteins along with human serum albumin, while more advanced drugs use proteins of human origin in a culture medium without albumin.

Modern protein production has successfully removed the addition of either animal or human proteins in the recipe [24].

One important area of research has been the creation of recombinant proteins with a longer half-life. This is because the infusion time required to maintain the minimum level of necessary clotting factors (>1%) is quite long, approximately 12 h for rFVIII and 16–18 h for rFIX, respectively [25]. This places an additional burden on patients, particularly children and older adults, and increases the cost of the procedure. The modification of coagulation factor molecules, such as PEGylation (a covalent modification with polyethylene glycol fragments) or “cross-linking” with FC fragments of antibodies, was proposed as a solution [26].

It is crucial to mention that these strategies that improve the drug’s half-life and lessen immunogenicity have been used in industrialized nations’ clinical settings. On the contrary, developing economies have not benefited from the aforementioned achievements due to the high cost of new procedures, leaving their patients with low quality of life [24]. Therefore, it is necessary to optimize replacement therapy to reduce costs and the number of interventions.

## 4. Non-Replacement Therapy for Hemophilia (Antibodies)

Antibody-based methods are being developed as an alternative to the extended half-life drugs mentioned above, which can significantly reduce the frequency of injections but still impose a lifelong burden of therapy on patients. Studies have demonstrated that the binding of specific antibodies to FVIII and IX leads to the activation of the coagulating factors. Antibodies also target inhibitors of coagulation processes, such as specific small interfering RNAs (siRNAs) or tissue factor pathway inhibitors (TFPIs).

The humanized bispecific monoclonal antibody, emicizumab, is transplanted onto human immunoglobulin (IgG4). By binding factors IX and X, it imitates the activity of FVIII and speeds up the process of their activation. Amazingly, emicizumab was designed to escape FVIII-neutralizing antibodies, which significantly increases its effectiveness even in their presence [27,28].

However, this drug works slightly differently from FVIII. When there is no bleeding, FVIII remains inactive, whereas emicizumab circulates in the bloodstream constantly in an “on” state. This significantly improves the plasma thromboplastin time, which affects the coagulation rates. This variation affects the degree of control over the drug’s effect and the results of diagnostic tests [29,30,31,32].

The frequency of emicizumab dosing during clinical trials was varied, with patients receiving the drug once per week, two weeks, or once in four weeks. The drug significantly reduced the number of bleedings compared to FVIII infusions when taken once every two weeks or once every four weeks. However, with more frequent use, side effects in the form of thrombosis or thrombotic microangiopathy (TMA) were observed [27]. In the study of 103 patients with hemophilia who received emicizumab prophylaxis, the cases of thrombotic microangiopathy and thrombosis were reported in 2 participants (1.94%). It is worth noting that these incidents occurred in patients after discontinuation of the activated prothrombin complex concentrate. This concentrate was administered for more than one day on average with a dosage of more than 100 U per kilogram daily [28]. In terms of immunogenicity, emicizumab’s performance is comparable to other humanized therapeutic monoclonal antibodies [33,34,35]. In a study by Roberts and his colleagues, it was shown that TFPI, a serine protease inhibitor, acts similarly on the factor VII (TF/FVIIa) activation pattern. TFPI exists in three isoforms [36], such as TFPIα, which is a protein found in plasma. This protein is made up of an acidic amino-terminal (N-) region, three tandem Kunitz domains, and intermediate peptides distributed between the Kunitz domains; TFPIβ was originally found in mice but has also been discovered on human cells, particularly ECV304, a human bladder cell line; TFPIδ is mostly expressed in the liver.

The first drug with anti-TFPI action was BAX499 (ARC19499), which contains Kunitz domains 1 and 3. However, it was unsuccessful and was rejected after the phase 1 clinical trial due to an increased number of bleeding incidents in subjects [37,38].

Another fairly well-known drug is concizumab, which has also entered medical practice. Concizumab was first approved in Canada in 2023 for the treatment of hemophilia B with FIX inhibitors for patients older than 12 years [39]. Later that year, concizumab was approved in Australia and Switzerland. The U.S., EU, and Japanese government authorities are considering approving concizumab trade [40]. The action is based on a humanized IgG4 antibody that specifically binds to the Kunitz-2 domain. The drug demonstrates a significant effect in restoring thrombin generation and initiating procoagulant effects [41,42,43]. In the placebo group, there were 24 cases of bleeding, while in the group taking the drug, bleeding was recorded only once.

However, there have been reports of drug-induced thrombosis in high-risk groups, such as older people and patients with concomitant cardiovascular complications, particularly when combined with other hemostatic drugs [44].

It is also worth mentioning other antibody-based drugs targeting TFPI, as listed in Table 1.

## 5. Cellular Therapy of Hemophilia

Hemophiliac patients who receive cell therapy have their endothelial progenitor cells and stem cells modified to express coagulation factors ex vivo before being transplanted into their bodies. Fibroblasts, adipocytes, and hepatocytes have also been successfully transformed for this purpose. This poses several difficulties, one of which is sustaining the transplanted cells’ blood clotting factor expression for a prolonged amount of time. Consequently, the main goals of the research are to identify the best cell type and provide techniques for cell transplantation in cell therapy.

A clinical trial study by Roth et al. was the first to report a statistically significant increase in blood clotting after cell administration. Six individuals with profound hemophilia A received 1–4 × 10^8^ fibroblasts that had been plasmid-transformed to express FVIII. The FVIII activity rose by about 1–5% in the group that received the maximum dosage of cells. But sustained expressiveness was not possible [6].

Using a retroviral or lentiviral vector, several studies have reported the successful transplantation of transformed hematopoietic stem cells that express FVIII or FIX. This has resulted in increased blood clotting levels over a long period, but only in animal models [53,54,55]. The potential of blood cells, such as platelets and red blood cells, to increase the expression of clotting factors has been the object of further research [56,57,58]. According to these studies, ectopically produced FVIII in transgenic mouse platelets seems to be resistant to circulating inhibitors [59,60]. This strategy has become a desirable therapeutic option for hemophilia A treatment. Furthermore, Omori and colleagues describe the use of a lentiviral vector to express a target protein through the transformation of hematopoietic stem cells driven by a promoter specific to platelets or megakaryocytes and then transplanting those cells into recipient mice [61,62]. Other studies demonstrate that inducing platelet expression of FVIII or activated coagulation factor VII, achieved by a similar method, causes a notable decrease in the incidence of bleeding in mice suffering from this illness [62,63,64]. There are also reports of crossing experimental animals with transgenic mice expressing FVIII under the control of a promoter known as Tie-2 [65]. It also increased FVIII activity and minimized the risk of bleeding.

**Figure 2:**
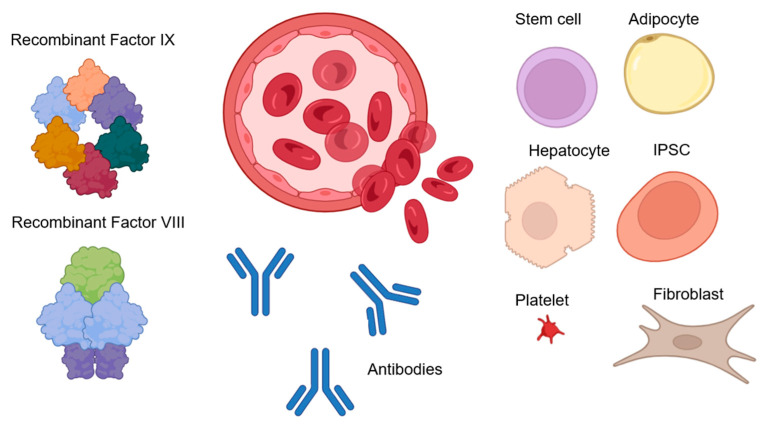
Replacement hemophilia therapies. Recombinant or purified clotting factors are frequently administered intravenously as part of therapy: FVIII and FIX. Using antibodies to encourage the activation of factors FVIII and FIX is another method of treating hemophilia as well as inhibiting coagulation processes. Cell therapy may also be a potential treatment for hemophilia, as a result of which endothelial progenitor cells, stem cells, fibroblasts, adipocytes, hepatocytes, platelets, erythrocytes, or iPSCs can be transformed to express blood clotting factors, and subsequent transplantation [53,54,55,56,61,62,66].

Advances in research on induced pluripotent stem cells (iPSCs) in the context of hemophilia treatment have also been made. This approach is attractive because the pool of cells expressing coagulation factors is more stable [66]. In their studies, Xu et al. obtained the first results of treating hemophilia using iPSCs [66]. In a study by Kashiwakura et al. [67], intraportal injection of iPS cells transformed with a lentiviral vector, after differentiation into endothelial cells, was found to improve bleeding rates in mice with hemophilia A. However, the duration of cellular expression and cell life after transplantation remains a problematic issue. To address this issue, 3D scaffolds and cell sheet technology have been employed [68,69]. These methods have enabled long-term gene expression and cell survival. For instance, when a cell sheet expressing FVIII was transplanted into mice with hemophilia A, stable expression of the clotting factor was observed for almost a year [70]. Figure 2 provides an overview of the various non-gene therapy treatments for hemophilia.

## 6. Gene Therapy of Hemophilia

Gene therapy stands out as a more viable treatment option despite the relatively extensive range of therapeutic alternatives available for hemophiliac patients. This is because gene therapeutics transfer a functional copy of the appropriate gene to induce endogenous synthesis of FVIII or FIX. Conversely, hemophilia is an excellent candidate for gene therapy since the disease’s clinical symptoms are brought on by the lack of a single protein, which must exist in trace amounts in the bloodstream. Many years of observational studies and clinical experience have demonstrated that the bleeding diathesis can be markedly altered by a small increase in circulating levels of deficient coagulation factors, from 1% to 5% of normal proteins. In other words, a significant result in the treatment of hemophilia can be achieved even with the low effectiveness of the drugs used. Plasma FVIII and FIX levels are reliable indicators of bleeding risk. These proteins can be easily measured using standard laboratory coagulation techniques. Transgene expression also does not require strict regulation of a specific level since the presence of FIX or FVIII in the blood can normally vary without causing toxicity. Animal models that have played an important role in the preclinical assessment of gene therapy approaches include dogs with hemophilia A and mice with FVIII and FIX knockouts [71,72]. Since plasma FVIII or FIX levels are highly correlated with bleeding risk, it is possible to evaluate the efficacy of therapy by taking their measurements.

### 6.1. Adeno-Associated Virus Vectors

Currently, one of the most efficient ways to introduce therapeutic genes into somatic target cells is through viral transduction, which is achieved using either naturally occurring or engineered virus vectors. Adeno-associated viral (AAV) vectors remain one of the preferred choices for gene delivery in a range of genetic diseases, including hemophilia. Seven AAV-based medications are currently in clinical use and have received marketing approval: Lumevoq—Leber Hereditary Optic Neuropathy (LHON), Luxturna—Leber congenital amaurosis (LCA), Zolgensma—spinal muscular atrophy (SMA), Glybera—lipoprotein lipase deficiency, ROCTAVIAN—hemophilia A, Hemgenix—hemophilia B, Elevidys—Duchenne cellular dystrophy, and BEQVEZ—hemophilia B [73,74,75]. AAV virus is thought to be non-pathogenic to humans with weak immunogenicity. It requires the help of a satellite virus, such as an adenovirus or herpesvirus, for its replication and productive infection. Among viral vectors, AAV vectors offer the best safety profile as a result of the aforementioned features. Recombinant AAV vectors do not contain the coding sequences of wild-type viruses, which reduces their likelihood of inducing a cell-mediated immune response to viral proteins. Even though the AAV2 serotype was the main focus of early gene therapy research, there are already over 100 naturally occurring AAV serotypes with different tropisms and immunobiological characteristics that can be employed in gene therapy applications. These vectors work well in dividing and non-dividing cells for transduction. The transgenes delivered by AAVs are easily manipulated, enhancing protein expression levels by adding stronger tissue-specific promoters and optimizing transgenic cDNA codons. The goal of codon optimization is to improve the translation efficiency of the AAV transgene by leveraging liver-derived high-expression domains, including the albumin gene. New synthetic capsids with greater potency and enhanced packaging capacity that specifically target tissues have been developed with bioengineered AAV vectors. Compared to other viral vectors, AAV vectors are smaller in packing size (~5 kb, including inverted terminal repeats). Nonetheless, a number of strategies have been developed to deliver a large therapeutic gene, such as employing a truncated gene that encodes a shortened but functional protein [73].

Due to the comparatively small coding region of FIX (1.5 kb) and significantly simpler translational pathway compared to FVIII, hemophilia B was the focus of early AAV-mediated gene therapy experiments. Katherine High’s team at the Philadelphia Hospital was the first to demonstrate therapeutic levels of FIX in circulation after AAV-mediated gene transduction [74]. This study aimed to mediate efficient gene delivery into the liver of patients who are suffering from hemophilia B by injecting AAV2 serotype vectors via the hepatic artery after selective catheterization. As observed in one of the study participants, plasma FIX levels increased to approximately 10% but then decreased to baseline values. Participants with the greatest dosage were observed to have a transgenic protein level drop to baseline accompanied by a transient, asymptomatic 10-fold elevation of liver transaminase activity. Subsequently, liver transaminases returned to baseline values within a few weeks, accompanying a decline in FIX levels. In animal models, including primates with administered doses ten times higher than the ones examined, there was no decrease in FIX expression linked to liver damage. Experts have proposed that a cytotoxic T-cell response specific to capsids that target transduced hepatocytes is likely the cause of this impact [74].

The first reliable findings of long-term FIX expression came from a University College London study conducted at St Jude Children’s Research Hospital. This study utilized the AAV8 serotype as a vector due to its special tropism for hepatocytes, which improves transduction during peripheral circulation administration and facilitates infusion procedures. Additionally, compared to AAV2, with over 70% immunoresistance in humans, the AAV8 serotype has a lower rate of immunoresistance of approximately ~25% [75].

In the field of gene therapy research, several strategies have been employed. These strategies include differences in the selection of AAV capsid, vector genome configuration, the expression cassette design, and viral vector production method (insect cell method–baculovirus versus mammalian system) [76]. In general, greater vector doses were needed for therapeutic transgene expression when vector preparations were generated by transducing insect cells with baculovirus [76].

AMT-061 (NCT03489291) was produced by UniQure by introducing a gain-of-function Padua mutation into the FIX sequence of AMT-060. Three patients with severe hemophilia B experienced a boost in FIX activity as a result, which was around eight times larger than previously recorded with AMT-060 at the same dose. In another study, 54 patients with moderate to severe hemophilia B in the U.S. and Europe were subsequently enrolled in an open-label phase 3 trial (HOPE-B, NCT03569891). After receiving a dose of 13 × 10^61^ vector genomes (vg) per kilogram of AMT-2, the average plasma level of FIX activity was 36.9% of normal 1.5 years later. As a result, there was a 64% drop in bleeding frequency and a 97% drop in yearly FIX use. These advantages were noted regardless of the presence of pre-existing anti-AAV5 antibodies [77]. AMT-061 is also known as the etranacogene dezaparvovek, which is a recombinant AAV serotype 5 containing a Padua optimized-codon variant of the factor IX transgene. The studies showed that the administration of AMT-061 led to a clinically significant increase in FIX activity, stopped bleeding, and suspended the need for FIX substitution. Usage of AMT-061 has led to a mean activity of IX factor increase of 47% within 26 weeks for three patients with initial FIX activity ≤ 1%. The advantage of etranacogene dezaparvovec is the absence of the need for immunosuppression and taking corticosteroids since patients did not experience an increase in the activity of liver enzymes. The advantage of etranacogene dezaparvovec is the absence of the necessity for immunosuppression, even in the initial presence of NAbs in patients. In addition, there is no necessity to take corticosteroids since patients did not experience an increase in the activity of liver enzymes [78].

Higher amounts of unmethylated cytidine phosphate guanosine (CpG) motifs in the FIX cDNA were found to reduce the activity levels of the transgenic FIX in a clinical investigation evaluating the FIX-R338L transgene in patients with hemophilia B. The theory is that an overabundance of unmethylated CpG motifs—which are prevalent in bacterial DNA but not in DNA from mammals—caused a reaction from Toll-like receptor 9. As a result, transduced hepatocytes were lost, along with related transaminitis that was resistant to corticosteroids [79].

In another study (SPK-9001, fidanacogene elaparvovec, NCT02484092), following a single vector dose of 5 × 10^11^ vg/kg, 15 patients showed a sustained mean FIX activity of 22.9% ± 9.9%. After injection, the annualized bleeding rate (ABR) was 0.4 ± 1.1, down from 8.9 ± 14.0 before gene therapy. The FIX-R338L transgene with fewer CpGs is present in a bioengineered capsid pseudotyped AAV vector that makes up SPK-9001 [80].

After a systemic dose of 5 × 10^12^ vg/kg BBM-H901, 10 hemophilia B patients showed steady-state FIX activity levels of 36.9% at 1 year in an investigator-initiated phase 1 research in China (NCT04135300) [81]. The Padua mutation is present in BBM-H901, a dimeric version of a codon-optimized FIX expression cassette that is controlled by a liver-specific promoter and enclosed in a modified liver-tropic AAV capsid. Prednisolone at a dose of 1 mg/kg for one week, followed by an eight-week dose reduction, was the preconditioning phase before the introduction of the vector. Two (20%) individuals had lower FIX activity and higher levels of ALT and aspartate aminotransferase [81].

Due to advancements in gene transfer techniques, plasma levels of FIX activity can now be sustained throughout an acceptable range. Nearly ten years ago, it was thought to be impossible for FIX activity to occur at normal physiological levels. However, modifications to the AAV vector have made this possible.

Fidanacogene elaparvovec (PrBEQVEZ™) was approved in 2023 for the treatment of hemophilia B by the Canadian Agency for Drugs and Technologies in Health (CADTH), later in 2024, the drug was approved by the U.S. FDA [82,83]. At the moment, Fidanacogene elaparvovec is under consideration by the European Union, and clinical trials are also being conducted in many countries. Beqvez is intended for patients over the age of 18 with factor IX activity of less than 2% who do not have neutralizing antibodies to AAVRh74var [84].

It should be noted that certain biological aspects of AAV vectors limit their widespread use. In a bioengineering context, the size-limiting capacity of AAV-based vectors (4.7 kb) is a significant obstacle, which complicates the cloning process of the target gene and optimizing the vector itself. Also, FVIII itself has a low expression profile, which makes using these kinds of vectors for hemophilia A gene therapy challenging. BioMarin, in their study [85], attempted to overcome these limitations. Deleting the FVIII B domain, which is not necessary for cofactor action, allowed the FVIII expression cassette to be smaller. Other research has used the strategy of rearranging the wild-type human FVIII cDNA to match the codon use of highly expressed human genes to increase FVIII expression tenfold [86,87].

Furthermore, 134 individuals with severe hemophilia A were included in the largest open-label, single-arm, multicenter, phase 3 clinical trial of gene therapy conducted by BioMarin to assess the created construct (BMN 270). The results showed a mean increase in levels of FVIII activity by 41.9 IU per deciliter between 49 and 52 weeks in 132 participants who tested negative for human immunodeficiency virus [88,89].

Another study conducted by Spark Therapeutics involved the dosing of 8011 patients with the SPK-18 construct. The human factor VIII gene has been codon-optimized in this AAV vector construct. The HEK-293 cell line-derived bioengineered LK03 capsid, which is pseudotyped with a liver-specific promoter, controls the gene. At the 33.4-month follow-up point on average (3.7–47.6), 16 of the 18 subjects maintained their FVIII expression. Two participants lost all expression, which is thought to be due to the effects of an increase in the level of ALT in blood. The program is presently undergoing phase 3 clinical trials [90].

Additionally, after AAV delivery, there is still a chance of liver damage due to the decrease or loss of transgene expression. Regardless of the transgenic promoter, production method, or AAV genome layout, transaminitis appears to occur with the majority of AAV capsids. In certain cases, corticosteroids used alone or in conjunction with other immunosuppressive medications can regulate this condition. Because this toxicity cannot be replicated in animal models, the pathophysiological basis of transaminitis is still unknown [91,92].

The episomal maintenance of proviral DNA is predominant; hence, the likelihood of insertional mutagenesis resulting from AAV-mediated gene transfer is generally regarded as minimal. This is consistent with the finding that, despite being common, human wild-type AAV infection is not associated with carcinogenesis. The study discovered that in a tiny proportion of human hepatocellular carcinoma samples, wild-type AAV2 genome fragments were integrated close to recognized proto-oncogenes [93]. Hepatocellular carcinoma, as a complication of AAV gene therapy, was observed in murine models but has not been detected in humans [94]. However, AAV2’s pathogenic function in this instance remains unknown.

It is noteworthy that neutralizing anti-AAV antibodies (NAbs) to specific AAV serotypes are pre-existing in 20% to 70% of patients and that these antibodies can impede effective gene transfer. Gene therapy trials currently do not accept patients with NAbs, which restricts the wide range of applications for gene therapy in hemophilia treatment. Serotype AAV17 is one tactic that has been effective in defeating NAbs in animal studies. However, because of the cross-reactivity of NAbs, this method might not work with humans [93].

It is also worth noting that when planning gene therapy using AAV vectors, it is important to take into account the characteristics of transduction in the context of histology, specifically the function of liver sinusoidal endothelial cells and Kupffer cells, which together constitute liver reticuloendothelial cells. With a diameter of 7 to 9 µm, sinusoidal endothelial cells are scavengers that can internalize particles as small as 0.23 µm in vivo when conditions are right. Kupffer cells with a diameter of 10 to 15 µm take in larger particles. Since the majority of gene transfer vectors are less than 0.23 μm in diameter, hepatocyte-directed gene transfer may be less effective if Kupffer cells and liver sinusoidal endothelial cells absorb the vectors [95].

### 6.2. Lentivirus Vectors

Compared to the AAVs described above, lentiviral vectors (LVs) are capable of their genome integration into host DNA, which is maintained during cell division. This may be advantageous for achieving long-term expression goals but may also carry additional risks of insertional mutagenesis [80,87]. Another advantage of these vectors is their low immunogenicity. Their ability to integrate into dividing and non-dividing cells makes them a desirable candidate for gene therapy [96,97].

The efficiency of a lentiviral vector for transducing human hepatic endothelial cells (HLECs) in vitro was examined in a study conducted by Totsugawa and coauthors [98]. The green fluorescent protein (GFP) gene was encoded in a pseudotyped lentiviral vector called LtV-GFP, which was created using the FuGENE 6 technique and enabled HLEC infection. After LtV-GFP infection at a multiplicity of infection of 10, around 95% of HLECs expressed GFP. Notably, LtV-GFP transduced HLECs maintained in vitro angiogenic potential in the Matrigel assay to the same degree as primary cultured HLEC. They also displayed gene expression of endothelial markers, including CD 34, factor VIII, flt-1, KDR/flk-1, and HGF. Finally, they had stable and long-lasting GFP expression. The findings of this study demonstrate that lentiviral vectors are a useful tool for cell and gene therapy [98].

A follow-up study by Niek P van Til and colleagues carried out on differential histological assessment of the efficiency of liver transduction with a lentiviral vector and an interesting clarification was identified: the use of gadolinium chloride to inhibit Kupffer cell function resulted in a significant decrease in GFP-positive non-parenchymal cells (2.15 ± 3.14%) and a seven-fold rise in GFP-positive hepatocytes (1.48 ± 2.01%) in comparison to naïve animals [99]. According to these findings, lentiviral transduction of hepatocytes is not substantially inhibited by sinusoidal endothelial cells; however, lentiviral particles are sequestered by Kupffer cells, which stops hepatocyte transduction. Therefore, the use of medications that block Kupffer cell function may be crucial for liver disease treatments based on lentiviral vectors.

In a different study, the authors investigated the effectiveness of lentiviral-mediated gene transduction in primary mouse LSEC utilizing reporter genes vs. plasmid-based techniques (lipofection, electroporation, and calcium phosphate) for in vitro gene transfer [100]. The outcomes demonstrate that, in comparison to lipofection and calcium phosphate transfection (6% and 4%, respectively), electroporation is the most successful in vitro plasmid gene transfer technique for delivering GFP to LSECs (31%). But in contrast to plasmid-based techniques, lentiviral transduction produced higher, more stable, and more efficient gene transfer (70%) [100].

Alternative microRNA (miR) medication was created in a study that successfully restored FIX activity in animal models of hemophilia B and stabilized transgene expression in the liver without showing any signs of genotoxicity [96]. The exact targeting of hepatocyte expression via a mix of transcriptional and post-transcriptional, miR-mediated regulation is necessary for the safety of these constructs [101]. Nevertheless, systemic administration of these medications to dogs has been linked to minor acute toxicity, and the treatments’ efficacy is modest at the levels used [101]. The study authors propose that this could be due to inadequate distribution of intravenously administered drugs to hepatocytes, potentially caused by rapid clearance from the circulation by professional phagocytes in the liver and spleen. This, in turn, triggers activation of the innate immune system upon perception of captured viral particles.

According to Shi et al., hematopoietic stem cells (HSCs) transduced with LV expressing FVIII may produce therapeutically relevant quantities of FVIII in mice treated with hyaluronic acid without developing antibodies. Glycosylation sites in the B domain were altered based on the codon-optimized F8-BDD construct to enhance FVIII secretion and function [102].

Hemophilia gene therapy has also made use of other LV vector designs. For example, in prior work, Shi et al. used an LV vector (2bF8 LV) containing the platelet-specific integrin alpha 2b promoter to express the FVIII gene, which was subsequently transduced into mouse bone marrow [103]. The mice that were administered the 2bF8 LV-transduced bone marrow showed correction of the hemophilia A phenotype, tail clipping survival, and functional FVIII activity. To produce long-term steady production of FIX in dogs with hemophilia B, a self-inactivating LV vector (SIN-LV), including a hepatocyte-specific promoter, was employed [104]. The 2bF9/MGMT LV vector, which carries the FIX, methylguanine DNA methyltransferase (MGMT) 140 K, and alpha-2b promoter, was used in another experiment. Following hematopoietic stem cell (HSC) transduction, the data demonstrated 3.7 times increased FIX activity and 2.9 times higher FIX expression in platelets [105]. The clotting time was statistically significantly reduced in the transplanted mice with elevated therapeutic platelet-FIX expression.

### 6.3. Gene Editing

A promising technique for genome editing and future gene therapy is the clustered regulatory short palindromic repeat (CRISPR)/CRISPR-associated (Cas) system [106]. In a study conducted by Huai et al. [107], genes in the F9 HB mutant mouse models were corrected using the CRISPR-Cas9 technology in both adult mice (in vivo) and germline cells (ex vivo). Hydrodynamic tail vein (HTV) injection was used to fix the mutation in the livers of HB mice by delivering the Cas9-sgRNA plasmid and donor DNA in vivo. The coagulation deficiency was reversed in 62.5% of mice treated with HTV, as evidenced by detectable gene correction (>1%) in the F9 alleles of their hepatocytes. Three distinct Cas9 variants were microinjected into HB mouse germline cells in an ex vivo investigation to examine their safety and effectiveness in gene repair [107].

Chen et al. [108] demonstrated the CRISPR-Cas9-mediated homology-independent integration of FIX at the albumin locus in a rat model of hemophilia B.

Many studies have shown the subtleties involved in utilizing CRISPR-Cas as a mechanism for modifying genes associated with hemophilia A or B. In these investigations, the clotting factor gene deficiency was either fixed in situ, or the strong albumin promoter was hijacked to drive FVIII or FIX by using the albumin locus as a haven [109]. Additionally, the findings showed how AAV integration fits into CRISPR/Cas-based gene editing. It is known that the genomes of AAV vectors are either randomly integrated into pre-existing double-strand breaks throughout the genome or episomal. AAV vector genomes, however, can also incorporate into particular CRISPR-induced double-strand breaks, according to current research [109,110]. Because integrated copies of AAV vectors containing CRISPR-Cas components result in continuous production of Cas9 and gRNA, this presents possible genotoxicity concerns. ITR-Seq, a next-generation sequencing technique, was created by Breton et al. [111] to identify in vivo AAV integration at genome-wide DNA editing sites. In preclinical and clinical investigations, this method may help to clarify the specificity and effectiveness of genome editing nucleases.

AAV is widely used in CRISPR-based research on mouse models as a vector to deliver CRISPR-Cas elements. On the other hand, extended expression of these genes raises the possibility of off-target events or DSB-induced side effects, such as chromothripsis or p53-mediated DNA damage responses [112,113].

A functional copy of the kFIX cDNA was incorporated by Intellia Therapeutics into the albumin “safe harbor” locus’ intron [114]. AAV encoding the F9 cDNA, CRISPR-Cas9 mRNA, and albumin guide RNA (gRNA) were delivered via non-viral lipid nanoparticles (LNPs). With the use of this LNP-AAV hybrid technique, accurate genome integration was made possible, leading to transient Cas9 expression and stable FIX expression driven by the endogenous Alb promoter, which decreased the possibility of off-target reductions [110].

Therefore, developing efficient non-viral gene delivery techniques is still necessary to introduce CRISPR-Cas components and a donor template into the target cells. A “hit and run” strategy would be ideal, in which CRISPR/Cas components are only present in transfected cells for a short period to accomplish effective gene editing, after which they are no longer required.

In Lee et al.’s [115] study, B was developed in a mouse model by using a combination of lipid nanoparticles and AAV to knock in the antithrombin gene using human FIX. The antithrombin (AT) gene was selected as the target locus for knock-in because it is involved in the anticoagulation pathway. The introduced insert was the coding sequence hFIX. Lipid nanoparticles carried components for CRISPR-Cas9 mutagenesis, and AAV-donor DNA sites ensured the formation of insertions or deletions in the gene encoding antithrombin after breaks were introduced into its DNA during the operation of the CRISPR-Cas9 system [115].

Lastly, the possibility that the production of the bacterial Cas9 protein, or any of its orthologs or derivatives, could set off an adverse immune response and eradicate gene-edited cells cannot be completely ruled out [116].

### 6.4. Non-Viral Delivery

Non-viral delivery refers to the use of vectors based on synthetic polymer or lipid particles (for example, liposomes and lipofectin). Such constructs act as packaging and protection for genetic material, ensuring its delivery into the cell. This approach has several advantages compared to viral vectors: theoretically, there are no restrictions on the size of the transferred genetic material, which allows operations with an immeasurably large number of proteins; higher safety in clinical use due to the absence of immunological complications; synthetic materials have a long shelf life; broad prospects for scalable production, reduced cost of final products.

However, such constructs may have difficulty penetrating cells and are also capable of expressing transgenes only for a limited period, at least at this stage of development of this technology.

To overcome these limitations, researchers have taken some experimental approaches; for example, hydrodynamic injection is used to increase the efficiency of the transfer of genetic material inside hepatocytes. By quickly injecting a sizable volume of fluid, this technique raises the liver’s venous pressure, which makes it easier for genetic material to enter hepatocytes [117,118]. There is a practice of using hydrodynamic injection in mice [119]. This does not exclude another problem: the fast reduction in transgene expression as a result of epigenetic silencing or genetic material loss in dividing cells.

In an attempt to solve this problem, researchers proposed a design called S/MAR (Scaffold Matrix Attachment Areas) [120]. It refers to the DNA sequences that, during interphase, connect chromatin to the nuclear matrix. DNA vectors that contain the S/MAR sequence allow for greater mitotic stability of dividing cells and avoid epigenetic silencing, subsequently leading to stable transgene expression. But even though this method was initially successful in maintaining transgenic expression in the livers of mice and pigs [121,122], S/MAR has not yet been tested in clinical trials.

Another approach is the use of lipid nanoparticles (DLNPs), the composition of which is as close as possible to cell membranes. They are generated by amphiphilic lipids, which can spontaneously form spherical structures with a hydrophilic interior when disseminated in an aqueous environment. The advantage of these structures is their high biocompatibility, virtual absence of immunogenicity and toxicity, as well as structural flexibility and ease of large-scale production. LNPs have been increasingly used in gene therapies as carriers for miRNA and mRNA [123].

Co-precipitation of calcium phosphate is a method for forming DNA nanoparticles. Plasmid DNA is co-precipitated with calcium phosphate and applied to cells for transfection [124]. Conversely, it is important to note that the transfection efficiency of these methods is low, and ongoing efforts are being made to improve the materials. One promising technology is based on cell receptor-mediated uptake [125]. Several studies have been conducted using non-viral gene therapy-based approaches in combined polymer biomaterials and nanomaterials technology, which is an emerging field [126].

One major safety concern for the transfer of non-viral DNA is the potential for endonucleases in physiological fluids and extracellular space to degrade the therapeutic gene. Encapsulating DNA in a nanocarrier may be a potential solution to prevent serum endonuclease degradation and increase circulation time [127]. Examples of nanocarriers that have been used are zwitterionic lipids, polyplexes (which are created by condensing negatively charged plasmid DNA with cationic polymers), and a mixture of cationic and neutral lipids with DNA [126].

Some studies also present the development of a new class of biodegradable polycations that can package a variety of genetic materials and transfect a wide range of cell lines. To obtain them, natural polysaccharides and oligoamines are used, which are converted into cationic polysaccharides with two to four amino groups. Reductive amination of oligoamino acids and intermittently oxidized polysaccharides are used to accomplish this. Additionally, treating cationic conjugates with ethidium bromide revealed that the majority of them could form stable complexes with plasmid DNA [128]. However, it is indicated that only polycations based on dextran–spermine were more effective for both in vivo and in vitro cell transfection [129].

Another study demonstrated the effect of an original design based on mRNA encoding FVIII encapsulated in lipid nanoparticles (LNPs). However, the injection of such a drug provides a relatively long-lasting expression of FVIII at a therapeutic dose (5–7 days), which may be useful for various applications in the treatment of hemophilia. There is a decrease in expression over time, but repeated injections of F8 LDL into immunodeficient mice resulted in persistent FVIII expression over time [130].

Developing non-viral vectors in vivo presents a challenge due to the accumulation in the target tissue and cellular internalization. Some delivery systems also form interactions with non-target cells, thereby creating negative feedback that affects delivery efficiency. Serum lipoproteins, for example, interact with some liposomal and small interfering RNA (siRNA) delivery systems [131]. Conversely, particles with strong positive surface charges [132] can experience unwanted aggregation.

To reduce nonspecific interactions, a common method is to use polyethylene glycol (PEG) to protect the interface of delivery vehicles [133]. Small interfering RNA (siRNA) delivery poses one of the most difficult challenges. The large size of mRNA coupled with its negatively charged and hydrophilic properties make it challenging for its movement across cell membranes. Hence, it is suggested that the use of nanocarriers and direct injection is necessary for the direct delivery of mRNA therapeutics into the cytosol of the target cells [134]. Like DNA, mRNA, and short double-stranded RNA, RNA also needs to be shielded from endo- and exo-ribonucleases that are found both within and outside of cells to prevent degradation [135]. Additionally, it is important to detect immune escape and endo/lysosomal escape [132], avoid nonspecific chemistry biomolecules or non-target cells, prevent liver clearance, allow extravasation to reach target tissues, and improve cell penetration.

A common issue with all gene therapy methods is their cost. Developing and implementing these procedures may be costly, especially in the beginning, to recover the research expenses. The main advantage of gene therapy as a clinical method is that it ensures the constant expression of endogenous coagulation factors, eliminating breakthrough bleeding and microbleeding. This reduces the need for repeated medical treatments and the likelihood of comorbidities, ultimately improving the standard of living. Gene therapy in the future can bring significant savings to the healthcare system of any country and increase the well-being of society as a whole. However, this may still be unaffordable for patients living in developing countries.

**Figure 3:**
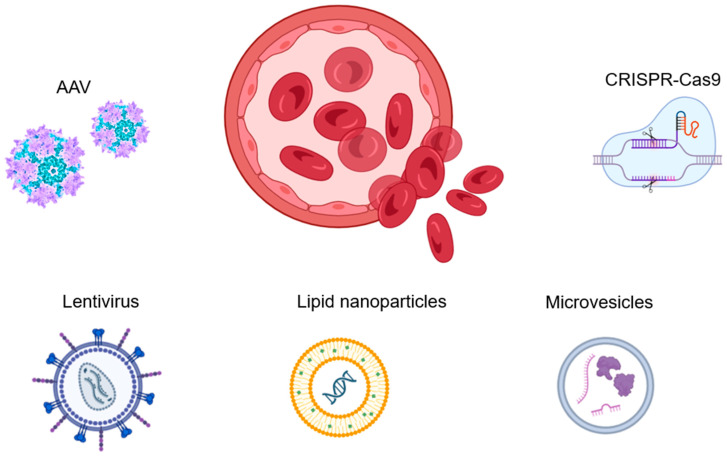
Gene therapy is the most promising and advanced treatment for hemophilia. AAVs are currently the preferred vectors for gene delivery, considering their safety and effectiveness. Lentiviral vectors can also be used, as they are embedded in DNA and supported by cell division, ensuring long-term expression. However, they may carry additional risks. The CRISPR system has significant potential in hemophilia therapy as an effective tool in genome editing. While non-viral vectors, such as synthetic polymers, biological nanoparticles, or microvesicles, can also be used in hemophilia treatment, their low efficiency of transfection and questionable safety of DNA delivery to the cell make them problematic. Currently, there is limited use of non-viral vectors.

Figure 3 summarizes the gene therapy agents and approaches used to treat hemophilia.

## 7. Clinical Applications of AAV-Based Gene Therapeutics for HEMOPHILIA A and B

In 2017, the first study of intravenous treatment of patients with hemophilia A using AAV-based liver gene therapy was reported. Due to its large size, the FVIII gene was packaged into an AAV viral vector that encoded the truncated version of the FVIII gene. The AAV capsid used in the vector genome configuration, the expression cassette design, and the vector production process (using a mammalian system as opposed to an insect cell or baculovirus) have all changed in subsequent gene therapy experiments (see Table 2). In general, when vector preparations were made via the insect–baculovirus approach, larger vector dosages were needed for therapeutic transgene expression. For instance, despite using a logarithmically higher vector dose of 2 × 10^13^ vg/kg, pseudotyped serotype AAV5 vectors (AMT-060; uniQure, Amsterdam, The Netherlands) made using the insect–cell–baculovirus method containing the same FIX gene cassette as in the St Jude-UCL study produced an average FIX activity level of 6.9%. Known cases of clinical research drugs for genetic therapy of hemophilia A and B are presented in Table 2 and Table 3.

Summarizing available clinical trial reports so far, it can be noted that in all clinically relevant current studies, different serotypes of AAV are used as a vector. However, the prevalence of antibodies against AAV capsid remains a problem, and the immunity to different AAV serotypes varies around the world, making it difficult to assess the effectiveness of drugs in different populations. This also creates the basis for the need to develop drugs based on other types of vectors (Lenti/CRISPR/non-viral).

## 8. Conclusions and Future Perspectives

There have been significant milestones already in the pursuit of hemophilia treatment through gene therapy. Patients with hemophilia A and B achieved significant clinical benefit when administered intravenously with AAV-based gene therapy (Table 2 and Table 3). Currently, severe hemophilia patients have been successfully transformed into mild hemophilia patients with the use of gene therapy. FVIII and FIX physiological levels have been achieved and remained within normal limits for a significant time. In most cases, bleeding has been limited or completely absent, even without prophylaxis intake.

However, there are still open questions and problems that require further research. The clinical effectiveness of gene therapy has been shown to increase in terms of long-term drug efficacy after a single intravenous infusion. Nevertheless, FVIII expression levels may decrease over time. One of the major challenges in gene therapy is immune resistance and hypersensitivity. As a result, gene therapy is currently unavailable for patients with antibodies to AAV, liver diseases, and severe concomitant pathology [76]. Although the efficiency of the expression level has been shown to increase, it is important to note that in some patients, it can vary from 0 to >200 IU/dL for unknown reasons. As a result, gene therapy is currently unavailable for patients with antibodies to AAV, liver diseases, and severe concomitant pathology. Although the efficiency of the expression level has been shown to increase, in some patients, it can vary from 0 to >200 IU/dL for unknown reasons. Regarding the issue of infusion toxicity, around 30% of patients experience infusion-related reactions, such as fever and hypotension, as well as unexplained liver dysfunction that occurs more than three months after infusion, which requires treatment with corticosteroids.

Research is currently being conducted in various RnD companies around the world, but gene therapy is still not available to many patients worldwide due to logistical reasons and high costs. There is a need for further study on the potential for the development of malignant neoplasms due to transfection and the possibility of limited integration of AAV into the patient’s genome.

Therefore, additional studies are required to address these concerns before gene therapy products are made publicly available. However, the potential for defeating hemophilia and developing treatments for other diseases is a constant motivation for large companies, government organizations, individual researchers, and laboratories worldwide. Gene therapy has a clear advantage over the use of recombinant purified FVIII and FIX, as well as antibodies, as it enables the long-term expression of transgenes. This will ultimately reduce the burden on healthcare in the future. An analysis by Machin et al. proposed that over 10 years, the total cost of gene therapy per person would be USD 1.0 million and result in 8.33 QALY (quality-adjusted life year), while prevention cost USD 1.7 million and resulted in 6.62 QALY [137]. The improvement of hemophilia therapy could potentially be achieved with the further developments of current approaches as well as the application of relatively new ones: the mutagenesis of recombinant FVIII and FIX to improve their properties, the creation of ribozymes with functional properties of FVIII and FIX, the creation of nanobodies with functional properties of the coagulant factors, using of nanoparticles to deliver mRNA of FVIII and FIX, editing of the genome to correct mutations in FVIII and FIX related to hemophilia, the increase in specificity of gene therapy, the use of a different combination of the above-mentioned approaches. Further development of gene therapy-related approaches for hemophilia will undoubtedly help reduce the cost of treatment and cover those who desperately need help now.

Nevertheless, gene therapy using AAV in the treatment of various diseases has several limitations and challenges. One of these problems is the prediction of human immunogenicity. Since AAV is introduced into the human body, an immune reaction occurs that prevents the widespread use of gene therapy. Innate and adaptive immunity are responsible for a specific and sustained response against AAV, limiting the efficiency of the re-administration of AAV-based drugs. Pre-existing AAV antibodies are a concern for the safety and efficacy of AAV vector-based gene transfer therapy. Recently, three deaths were reported in children treated with AAV-8 expressing the MTM1 gene for XLMTM (NCT03199469), and the clinical trial was suspended until the cause of death could be determined [138].

Unfortunately, preclinical studies are still unable to predict and fathom the complexity of the immune response in clinical trials. To solve this problem, several elegant solutions have been proposed to mitigate the risk of AAV-based gene therapy. Innate immunity can be tuned and silenced to prevent hypersensitive DNA recognition, and TLR activation often occurs after AAV administration. Moreover, transgene sequence and AAV expression cassettes can be designed to avoid innate response activation [139]. Additional optimization strategies may also include evasion from the complement system or reduction in transgenic expression in antigen-presenting cells (APCs). Humoral immunity can be shaped toward avoiding neutralizing antibodies (NAbs) formation by modifying AAV [140,141]. Moreover, cell-mediated immune responses can be tuned by using systemic immune suppression reduced capsid representation or a Treg-based strategy. Alternatively, transgene immune responses can also be turned off through the use of systemic immune suppression or Treg-based strategy or targeted delivery to tissues [142].

Another issue that needs to be solved for the next-generation gene therapies for hemophilia is first-in-patient dose prediction and selection. To achieve high efficiency of gene delivery to targeted cells using AAV vectors, the dosing regimen should be carefully established. There is no doubt that low doses of AAV may not provide a sustained level of gene expression and may be unlikely to transduce a clinically relevant number of cells to alter disease progression. On the other hand, high doses of AAV may result in transduction-related toxicities [143]. In addition, the administration of large amounts of AAV particles increases the risk of off-target transduction of neighboring cells, causing “bystander effects” [144,145]. This problem must be solved in order to conduct clinical trials in humans by choosing the optimal dose of AAV, which will not cause major side effects and will have a clinically significant effect. There are several main approaches in determining first-in-patient (FIP) doses for AAV gene therapy: allometric scaling of the gene efficiency coefficient (log GEF) and body weight (log W), the body-weight-based direct conversion method and allometric scaling of the gene efficiency coefficient (log GEF) and W-0.25. As a result of preclinical and clinical trials for 9 different AAV vectors, the disadvantages and advantages of these methods for calculating FIP doses were discovered. Overestimation of first-in-patient doses occurred with allopatric scaling between the gene efficiency coefficient (log GIF) and body weight (log W). An underestimation of first-in-patient doses was observed when using the body-weight-based direct conversion method. The most successful model for calculating FIP doses was using allometric scaling of the gene efficiency coefficient (log GEF) and W-0.25 [146].

For patients with pre-existing anti-AAV antibodies, repeated administration of AAV for a gene-therapeutic effect is a debatable moment. Today, the identification of total or neutralizing anti-AAV antibodies is a mandatory requirement for efficient and safe gene therapy with AAV vectors. If gene therapy is prescribed but AAV serotype-specific antibodies are detected, several approaches (e.g., plasmapheresis and enzymatic IgG degradation) have been developed to circumvent this limitation [147,148]. Clinically relevant antibody thresholds (total Ab or NAbs only) and quantification protocols should be clearly defined to avoid adverse effects of newly developed AAV-based therapeutics. Therefore, it is believed that the repeated administration of a drug containing AAV for such patients will be possible with the help of plasmapheresis. As a result of studies conducted on primates, with repeated administration of AAV after two or three plasmapheresis procedures, a decrease in the titer of antibodies to AAV was noted. With repeated administration of AAV, the antibody level returned to an elevated level [149]. Another study evaluated the effectiveness of transgene expression in animals with existing antibodies to the vector, treated with AAV with and without preliminary plasmapheresis. Thus, for animals with previously performed plasmapheresis, a high level of transduction of 60.8 ± 18.0% was observed compared with animals for which plasmapheresis with a transduction level of 10.1 ± 6.0% was not performed [150].

Plasmapheresis is not the only method of removal of antibodies to AAV for repeated administration. Neutralizing antibodies (NAbs) that appear in response to the introduction of AAV make it difficult to readminister viral vectors. It is associated with T and B cells immune activation and the B7/CD28 and CD40/CD40L signaling pathways. In Xiao Xiao and colleagues’ study, mice were injected with AAV carrying CTLA4Ig (cytotoxic antigen 4 associated with T lymphocytes) and CD40Ig for an immune-suppressing effect. Both CTLA4Ig and CD40Ig transported AAV reduced the number of NAbs and suppressed the activation of T and B cells, thereby allowing the reintroduction of AAV for gene therapy. This method is a promising solution to the problem of reintroduction of AAV [151].

Although AAV gene therapy for hemophilia is currently one of the most expensive drugs ever developed, there is no doubt that technological advances will soon help to scale up the manufacturing process for AAV-based drugs and minimize their cost. The delivery of a low copy (low dose) AAV vector to provide lifelong expression of a therapeutic gene (FVIII and FIX) in a physiological range is a major goal for the next generation of gene therapeutics. We also anticipate that new viral or non-viral vehicles will be developed and proposed for site-specific delivery of a transgene. We also foresee that AAV-based gene therapy for hemophilia will become more personalized according to the patient’s immune background and disease phenotype, which will help to reduce the potential risks and adverse effects of future gene therapy.

## Figures and Tables

**Table 1:** Recombinant monoclonal antibodies for hemophilia treatment.

Antibody and Producer	Description
**Marsticimab** **(Pfizer, Brussels, Belguim, Cambridge, MA, USA)**	This drug is a humanized IgG1 monoclonal antibody that binds to the Kunitz-2 domain of the TF/FVIIa complex in the extrinsic route of the coagulation cascade. With promising results (a considerable decrease in the number of bleeding in a group of 42 volunteers), the medication passed the first round of clinical trials, allowing for the continuation of research [45].
**Befovacimab** **(BAY 1093884)** **(Bayer, São Paulo, Brazil, Berlin, Germany)**	Monoclonal antibody IgG2 that binds to Kunitz domains 1 and 2. In 2018, positive results from a multicenter, open-label, phase 1 research with patients with hemophilia A and hemophilia B (both with and without an inhibitor) were presented. Preclinical and clinical trial data were encouraging at first, but befovacimab’s suitable safety profile was not later verified. Concerns regarding the predictability of thrombosis following befovacimab treatment are raised by the absence of laboratory abnormalities linked to SAEs or differential PK/PD characteristics in participants experiencing SAEs. This underscores the necessity for additional research on the therapeutic window of anti-TFPI treatment [46].
**MG1113** **(Greencross, Gyeonggi-do, Korea)**	The drug was designed based on the IgG4 monoclonal antibody’s ability to bind Kunitz domain 2. It is used through both intravenous and subcutaneous administration. Results from in vitro and in vivo investigations in animal models of the ongoing phase 1 trials have demonstrated restoration of thrombin production and a decrease in bleeding [47,48].
**Mim8** **(Novo Nordisk, Måløv, Denmark)**	The Duobody platform was used in the development of Mim8 [49] to screen for anti-FX and anti-FIXa antibodies. It is a new generation of bispecific antibody for the treatment of hemophilia A disease (by subcutaneous injection). The medication demonstrated low immunogenicity, low viscosity, enhanced activation on the membrane surface, and low binding of FIX and FX in solution in laboratory testing [50]. Despite having a distinct chemical structure, mim8 shares the bridging function of FIXa and FX with emicizumab. For instance, the emicizumab group and the anti-FIXa Mim8 group identify distinct epitopes [51]. Clinical trials in phases 1 and 2 are presently being conducted to assess the safety and efficacy of Mim8 in vivo [52].

**Table 2:** AAV-based gene therapies for hemophilia A.

Name	Company	Description
ASC618	ASC Therapeutics, Milpitas, CA, USA	Clinical phase: 2 The drug was designed using an AAV8 vector, which encodes the B domain of codon-optimized human factor VIII under a synthetic liver-directed promoter intended for liver expression (NCT04676048).
ANB-010	BIOCAD, Saint Petersburg, Russia	Clinical phase: 2 The drug uses an AAV vector encoding human FVIII. Work on the investigational drugs has been ongoing since the beginning of 2018, and the first experiments to evaluate the effectiveness on animals were started in 2019. On 26 May 2023, the Ministry of Health of the Russian Federation issued permission to conduct a clinical trial ANB-010-1/EDELWEISS “Assessment of safety, pharmacodynamics, biodistribution, immunogenicity and effectiveness of the drug ANB-010 in patients with hemophilia A”.
BMN 270: valoctocogene roxaparvovec (ROCTAVIAN™)	BioMarin Pharmaceutical, Novato, CA, USA	Clinical phase: 3 BMN 270 uses adeno-associated viruses (AAV 5) as carrier genes to express the protein factor VIII via a liver-selective promoter, which is lacking in people with hemophilia A. In this phase 3 open-label study, 54 men with hemophilia B (Factor IX activity ≤ 2% of normal) received a single infusion of adeno-associated virus 5 (AAV5) vector expressing the Padua variant of factor IX (etranacogene dezaparvovec; 2 × 10^13^ genome copies per kilogram of body weight), independent of pre-existing AAV5 neutralizing antibodies. This was done after a run-in period of prophylaxis with factor IX (NCT03569891). In the run-in phase, the annualized bleeding rate was 4.19 (95% confidence interval [CI], 3.22 to 5.45); for 7 to 18 months following therapy, it dropped to 1.51 (95% CI, 0.81 to 2.82), resulting in a rate ratio of 0.36 (95% Wald CI, 0.20 to 0.64; *p* < 0.001). The least-square mean of factor IX activity increased from baseline by 36.2 percentage points (95% CI 31.4 to 41.0) at 6 months and by 34.3 percentage points (95% CI 29.5 to 39.1) 18 months after treatment. During the posttreatment period, participants’ average annual usage of factor IX concentrate decreased by 248,825 IU (*p* < 0.001 for all three comparisons). Participants with pre-dose AAV5 neutralizing antibody titers of less than 700 showed benefit and safety. At the end of the study, no significant side effects connected to the medication were observed [136]. The European Medicines Agency (EMA) gave conditional approval to ROCTAVIAN in August 2022. Following this announcement, BioMarin announced the cost of the drug at EUR 1.5 million. In June 2023, the U.S. FDA approved ROCTAVIAN for the treatment of hemophilia A in adults with severe cases without pre-existing adeno-associated virus serotype 5 antibodies detected by an FDA-approved test.
PF-07055480	Pfizer, New York, NY, USA (formerly developed by Sangamo Therapeutics)	Clinical phase: 2 Hyroctocogen fitelparvovec is designed to deliver a shorter but working version of the *F8 gene* to hepatic cells, which are responsible for clotting factors synthesis in the body. The gene is packaged in SB-525 (PF-07055480) vector, which encodes liver-specific promoter, and AAV2/6 with improved liver tropism serves as a delivery mechanism to these cells (NCT03061201).
SPK-8011/RG6357	Roche, Basel, Switzerland (in collaboration with Spark Therapeutics)	Clinical phase: 2 SPK-8011 is an AAV-based (AAV-LK 03) capsid-expressing human FVIII. In July 2021, investigators presented information on a phase 2 study of SPK-8001, a new bioengineered AAV vector using the AAV-LK 03 capsid, also called Spark 200. The investigational SPK-8011 was dosed to 17 subjects in the phase 2 study: 2 at a dose of 5 × 10^11^ vg/kg, 3 at a dose of 1 × 10^12^ vg/kg, and 9 at a dose of 2 × 10^12^ vg/kg. Due to potential cellular immunity in response to the AAV capsid, two subjects experienced a loss of FVIII expression. After a two-year follow-up, eleven out of the fifteen participants who still had FVIII expression showed no significant decrease in FVIII activity (mean 12.6 ± 7.3% of normal at 26–52 weeks compared with 11.8 ± 7.2% of normal 52 weeks after vector injection; 95%, range: [−1.9, 0.3]) (NCT 03432520).
BBM-H803	Belief BioMed, Shanghai, China	Clinical phase: 1 BBM-H803, also called BBM-002, is designed to deliver a working version of *F8* into liver cells using a harmless AAV vector. Once the *F8* gene is unloaded from the viral vector, liver cells can begin to produce FVIII. This is expected to increase FVIII levels over a long period, thereby helping to prevent bleeding (NCT05454774).
SB-525	Pfizer (formerly developed by Sangamo Biosciences)	Clinical phase: 3 SB-525 is a gene therapy that uses a recombinant adeno-associated virus vector 2/6 (AAV 2/6) encoding a cDNA for the deleted B domain of human FVIII. The Alta trial is a phase II single-dose, dose-ranging study. Hyroctocogene fitelparvovec is a recombinant vector of AAV serotype 6 (rAAV 6), often referred to as SB-525 and PF-07055480. Four groups of two patients each received four ascending doses of gyroctcogene fitelparvovec (9 × 10^11^, 2 × 10^12^, 1 × 10^13^, and 3 × 10^13^ vg/kg). The dosage group was subsequently extended to 3 × 10^13^ vg/kg by adding three more patients. Eleven of the participants in this study were male. Patient follow-up lasted from 35 to 144 weeks; one patient from group 1 × 10^13^ vg/kg was later withdrawn from the study. In summary, elevated alanine aminotransferase (ALT; 8 [72.7%]), elevated aspartate aminotransferase (AST; 5 [45.5%]), upper respiratory tract infections (4 [36.4%]), and fever (4 [36.4%]) were the most frequently reported adverse effects. One patient (from the 3 × 10^13^ vg/kg dose cohort) reported treatment-related complications, hypotension, and fever within ≈6 h of drug infusion. The increase in factor VIII activity from baseline was generally successful but relatively short-lived. Patients in cohort 3 × 10^13^ vg/kg maintained a mean value in the normal range for factor VIII activity for five weeks after infusion, with a mean FVIII activity value of 63.5. About 3 weeks post-gyroctocogen prophylaxis administration, no bleeding was observed in any patient treated in the 3 × 10^13^ vg/kg cohort (NCT 04370054).

**Table 3:** Gene therapy for hemophilia B.

Name	Company	Description
ANB-002	BIOCAD	Clinical phase: 2 A new gene therapy for the treatment of hemophilia B based on BIOCAD proprietary AAV capsid vector. It contains an expression cassette—a synthetic DNA fragment encoding the therapeutic gene for human blood clotting factor IX (FIX). On 20 February 2023, the Russian Ministry of Health issued permission to conduct the clinical trial ANB-002-1/SAFRAN.
PF-06838435 (fidanacogene elaparvovec)	Pfizer	Clinical phase: 3 A single dose of this medicine is administered intravenously (IV) to prevent frequent bleeding episodes in patients. Paduya‘s working F 9 gene is packaged in an engineered delivery vector called adeno-associated virus (AAV)-Spark 100. It is approved as Beqvez for adults with moderate to severe hemophilia B who tested negative for antibodies against the viral vector therapy. The safety and pharmacological properties of fidanacogene elaparvovec were initially studied in an open-label phase 1/2a study (NCT02484092). Following promising results from the phase 1/2a study, a phase 3 clinical trial called BENEGENE-2 (NCT03861273) was initiated to study gene therapy in a larger population of patients with hemophilia B. Results from the first 45 participants showed a 71% decrease in bleeding rate after gene therapy treatment compared with the run-in period (1.3 vs. 4.43 bleedings per year). The rate of bleeding requiring treatment decreased by 78%, while the rate of replacement therapy infusions decreased by 92%. Two years after treatment, the average activity of FIX in treated individuals was 25% normal. In comparison, FIX activity of 5% to 40% is considered a mild form of hemophilia B. Patients with this percentage activity often only have noticeable bleeding from trauma or surgery, not spontaneous bleeding. Participants will be assessed over 15 years to assess long-term safety outcomes.
Idanacogene elaparvovec	Pfizer/Spark Therapeutics, Philadelphia, PA, USA	Clinical phase: 2 At a dosage of 5 × 10^11^ mg/kg, the hepatotropic bioengineered medication fidanacogene elaparvovec, which is based on the AAV vector and effectively delivers transgenes, has demonstrated sustained FIX levels and a low average annual bleeding rate. For 45 adult male volunteers with moderate to severe hemophilia B, Pfizer started a phase 3 open-label study to evaluate the safety and effectiveness of factor IX (FIX) gene transfer using PF-06838435 (Raav-Spark 100-Hfix-Padua). Pfizer reported the results at the February session of the EHAAD 2023 conference. No bleeding incidents occurred in 64.5% (29/45) of the participants between week 12 and month 15 following the medication infusion. In months 15 and 24, the average FIX activity was 27.5% and 25%, respectively. The majority of patients, over 80%, had FIX levels higher than 5%. In total, 62.2% (28/45) of the subjects were administered corticosteroids. The corticosteroid treatment lasted an average of 107 days. No corticosteroids had been administered to any of the participants in the first year after the drug infusion (NCT 02484092).
BBM-H901	Belief BioMed	Clinical phase: 1 An AAV vector is used to deliver the FIX Padua gene. Ten adult men in China participated in an open-label, phase 1 research (NCT04135390) to evaluate the safety and efficacy of a single intravenous (IV) infusion of BBM-H901. The pretreatment FIX activity of less than two international units per deciliter of blood (UI/dL), the absence of FIX protein antibodies, and low levels of AAV-specific antibodies were present in the eligible participants. Following a week of consistent doses of the immunosuppressive drug prednisolone, patients received a single dosage of BBM-H901 at a rate of 5 × 10^10^ vector genomes per kilogram of body weight (vg/kg). There was a reduction in the median number of target joints from 1.5 to 0, in the median annualized bleeding rate from 12 to 0, and in the median number of FIX replacement therapy infusions from 53.5 to 0. It should be noted that the target joints are the joints where bleeding often occurs.
VGB-R04	Shanghai Vitalgen BioPharma Co, Shanghai, China	Clinical phase: 1/2 VGB-R04 is a novel AAV vector armed with a high specific activity variant of factor IX. Volunteers are currently being recruited for clinical trials. Six weeks before the injection of VGB-R04, each participant in this study will give their informed consent and go through a screening evaluation. Each participant will undergo preliminary safety monitoring for 52 (±2) weeks and be allowed to take part in a follow-up study to assess VGB-R04’s long-term safety for five years.
AskBio009	Takeda, Cambridge, MA, USA	Clinical phase: 2 The liver-specific transthyretin promoter/enhancers, an AAV2-derived inverted terminal repeat, and codon-optimized complementary DNA (cDNA) encoding the hyperactive FIX (R338L) Padua variant were all included on the BAX 335 expression cassette. This allowed for the delivery of FIX (NCT01687608)
AMT061 (etranacogene dezaparvovec)	uniQure, Amsterdam, The Netherlands/CSL Behring, King of Prussia, PA, USA	Clinical phase: 3 AMT-061 consists of Padova-type FIX, a mutant gene with eight to nine times the expression of wild-type FIX, and LP 1, under the control of a liver-specific promoter, within the AAV 5 viral vector. The latest report of the phase 3 etranacogene desaparvovec trial in February 2023 was by CSL Behring at the EHAAD meeting. According to the report, 96% (52/54) of participants achieved a sustainable FIX level. In one patient, therapy was discontinued due to an allergic reaction. The second patient who did not benefit from the trial had a very high level of antibodies against AAV. High levels of antibodies to the AAV vector were present in 39% (21/54) of the subjects. Twenty of them were able to produce robust expression of FIX with titers less than 700 (NCT 03569891). The average annual bleeding rate for months from 7 to 24 was recorded to be no more than 1. In the 18th month, the average FIX expression activity was 36.9%, whereas in the 24th month, it was 36.7%. After the infusion, ALT (alanine aminotransferase) levels increased in 21% (11/52) of subjects. Of the 52, 17% were administered corticosteroids. The corticosteroid treatment lasted for eighty days. Corticosteroids were discontinued for all the participants after the 6th month. Hepatocellular carcinoma (HCC) was discovered in one patient a year following the infusion. In December 2020, the FDA originally put an end to the etranacogene desaparvovec investigation. Following months of investigation, UniQure determined that the treatment was “very unlikely” to result in cancer. In February 2023, etranacogene dezaparvovec received conditional clearance from the European Medicines Agency. November 2022 saw the U.S. FDA approve Etra. Shortly after, CSL declared the price of this drug to be USD 3.5 million.
